# Perceptions, risk and understandings of the COVID-19 pandemic in urban South Africa

**DOI:** 10.4102/sajpsychiatry.27i0.1580

**Published:** 2021-06-28

**Authors:** Andrew W. Kim, Raquel Burgess, Nicola Chiwandire, Zwannda Kwinda, Alexander C. Tsai, Shane A. Norris, Emily Mendenhall

**Affiliations:** 1 SAMRC/Wits Developmental Pathways for Health Research Unit, Faculty of Health Sciences, University of the Witwatersrand, Johannesburg, South Africa; 2 Center for Global Health, Massachusetts General Hospital, Boston, Massachusetts, United States of America; 3 Harvard Medical School, Harvard University, Boston, Massachusetts, United States of America; 4 Department of Social and Behavioral Sciences, Yale School of Public Health, Yale University, New Haven, Connecticut, United States of America; 5 Department of Epidemiology and Biostatistics, Faculty of Health Sciences, University of the Witwatersrand, Johannesburg, South Africa; 6 The Mongan Institute, Massachusetts General Hospital, Boston, Massachusetts, United States of America; 7 Science, Technology, and International Affairs Program, Edmund A. Walsh School of Foreign Service, Georgetown University, Washington, District of Columbia, United States of America

**Keywords:** COVID-19, perceptions, risk, knowledge, South Africa

## Abstract

**Background:**

How people perceive the coronavirus disease 2019 (COVID-19) pandemic and understand their risk can influence their health, behaviours and overall livelihood. The disease’s novelty and severity have elicited a range of attitudes and perspectives countrywide, which consequently influence the public’s adherence to public health prevention and treatment guidelines.

**Aim:**

To investigate perceptions, experiences and knowledge on COVID-19 in a community-based cohort study.

**Setting:**

Adults living in Soweto in South Africa’s Gauteng province during the first six weeks of the national lockdown regulations (i.e. Alert Level 5 lockdown from end of March to beginning of May 2020).

**Methods:**

Participants completed a series of surveys and answered open-ended questions through telephonic interviews (*n* = 391). We queried their perceptions of the origins of COVID-19, understandings of the disease, personal and communal risks and its relations with the existing disease burden.

**Results:**

Findings from our sample of 391 adults show that perceptions and knowledge of COVID-19 vary across several demographic characteristics. We report moderate levels of understanding about COVID-19, prevention methods and risk, as well as exposure to major physical, psychosocial and financial stressors. Depressive symptoms, perceived infection risk and concern about COVID-19 significantly predicted COVID-19 prevention knowledge.

**Conclusion:**

Public health communication campaigns should focus on continuing to improve knowledge and reduce misinformation associated with the virus. Policymakers should consider the mental health- and non-health-related impact of the pandemic on their citizens in order to curb the pandemic in a manner that maximises well-being.

## Introduction

How individuals perceive and experience the coronavirus disease 2019 (COVID-19) and understand their risk of infection can have major impacts on their health behaviours, risk of current and future diseases and overall livelihood.^1,2,3^ The novelty and severity of the disease have generated a diverse array of perceptions and experiences countrywide, which may consequently influence the public’s adherence to public health guidelines on prevention and treatment.^4,5,6^ The relationship between perceptions and preventative behaviour is in accordance with the established models of behaviour change,^7^ which indicate that an individual’s perceived susceptibility and severity of a health issue are key components that shape the attitudes towards diseases and behavioural modification.^8,9^ The associations between individual perceptions and health behaviours are further supported by the existing research on HIV in South Africa and elsewhere and the associations between exposure to information about HIV and sexual risk behaviours.^10,11,12^

Growing evidence of COVID-19 perceptions and experience in South Africa suggests elevated levels of pandemic-related social adversity and the risk of infection during the pandemic and subsequent lockdown regulation.

From 26 March 2020 until 30 April 2020, the South African government imposed a strict ‘national lockdown’ policy (Alert Level 5) to mitigate the risk of COVID-19 spread, which barred individuals from leaving a strict quarantine except for food, medicine and essential labour. Recent cross-sectional analyses of national public perceptions on the COVID-19 pandemic conducted by the Human Sciences Research Council suggest that a majority of survey respondents adhered to regulations, about one in five perceived their risk of infection to be high and an alarming portion of individuals experienced financial hardship and food insecurity, particularly amongst those living in informal settlements.^6^ Data from the recent National Income Dynamics Study – Coronavirus Rapid Mobile Survey illustrate a significant positive association between perceived risk and engagement in the preventative behaviour for COVID-19 amongst their sample of 7074 South African adults.^13^ We extend these national-level study to provide deeper understandings of the lived experience of the COVID-19 pandemic in a concentrated sample of adults living in Soweto to inform the development and implementation of ongoing public health campaigns aimed at reducing the burden of the growing COVID-19 pandemic in South Africa and across the world.

This study investigated the perceptions, experiences and knowledge of COVID-19 in a community-based cohort study of adults living in Soweto located in the Gauteng province during the first 6 weeks of the country’s nationwide lockdown policy (i.e. late March to early May 2020). Firstly, we characterised the diverse understandings of the disease across key demographic factors and then qualitatively described the most frequent and salient perspectives that emerged from our mixed-method interviews with 391 adults. Secondly, we examined the possible demographic, psychological and social predictors of knowledge on COVID-19. We have previously described the mental health impacts of COVID-19 experiences in an analysis amongst the same sample and found that greater levels of perceived risk of COVID-19 infection were associated with greater depressive symptoms, particularly amongst adults with greater histories of childhood trauma.^2^ Greater understanding of COVID-19 perceptions and experiences and identification of the primary drivers of community awareness of the disease can help to identify potential areas of intervention for effectively raising awareness about COVID-19 prevention as well as facilitate insight into the health and social challenges that South Africans are facing as a result of the pandemic. Ultimately, these insights could help to strengthen national efforts to contain the pandemic across South Africa and mitigate other important consequences of the pandemic on South Africans’ well-being.

## Methods

### Study setting

The research in this study was nested within the Developmental Pathways for Health Research Unit (DPHRU), a research unit associated with the South African Medical Research Council and the University of the Witwatersrand and located at Chris Hani Baragwanath Academic Hospital in Soweto, South Africa. All research participants were residents of Soweto, a predominantly black African urban township located southwest of the greater Johannesburg region. Amongst more than 1 million people living in Soweto, there is an elevated affliction of two infectious conditions such as HIV and tuberculosis (TB), amongst many others^14^; non-communicable diseases, such as hypertension, type 2 diabetes and depression^15^; and adverse social conditions such as poverty, unemployment and high levels of everyday violence.^16,17^ These conditions are further compounded by costly healthcare services in the private sector and systemic barriers to the public sector.^18^ For these reasons, we were interested in understanding whether people perceived their risk for COVID-19 to be higher than those in the general population, given the elevated rates of conditions such as hypertension, diabetes and HIV.

### Sample characteristics

Individuals interviewed in this study were selected from two studies pre-existing the COVID-19 outbreak and lockdown. One sample came from an epidemiological surveillance study of comorbidities, including mental, infectious and cardiometabolic diseases, whilst another source of data came from a longitudinal birth cohort study assessing the mental health impacts of intergenerational trauma. In the epidemiological surveillance study of comorbidities, participants were first enrolled into the study between April 2019 and March 2020. Data collection took place at their homes. Participants from this study were recruited based on a simple random sample of geographic coordinates within the boundaries of Soweto (*n* = 957). The second study sampled individuals already enrolled in the existing cohort study, which took place between January and March 2020 (*n* = 100). Research staff followed up on these participants to conduct follow-up data collection on COVID-19 experiences.

All research participants from both studies were residents of Soweto, were 25 years or older and completed two waves of data collection: the first wave was achieved in person, which took place before the COVID-19 pandemic, and the second wave of data collection happened telephonically during the Alert Level 5 lockdown between late March and early May 2020. The pooled sample represented a wide range of age and socio-economic status (SES), although a majority of our total pooled samples were women (Table 1).

**TABLE 1:** Socio-demographic characteristics of the sample (*n* = 391).

Sociodemographic characteristic	*N*	%	Mean	s.d.
**Age**				
25–30	122	31.8	-	-
31–44	103	26.8	-	-
45–54	63	16.4	-	-
55–64	61	15.9	-	-
≥ 65	35	9.1	-	-
**Gender**				
Female	278	71.8	-	-
Male	109	28.2	-	-
**Education**				
No school or primary school	194	50.1	-	-
Secondary school	132	34.1	-	-
Professional/teaching/university	51	13.2	-	-
Other	10	2.6	-	-
**Household density**				
Household assets	-	-	7.87	1.8
**Have you heard of coronavirus?**				
Yes	390	100	-	-
No	0	0	-	-
**Have you ever tested for COVID-19?**				
Yes	5	1.3	-	-
No	381	98.7	-	-
**If yes, what was the result?**				
Positive	0	0	-	-
Negative	6	100	-	-
**Perceived COVID-19 infection risk**				
Less risk than others	171	44.7	-	-
Same risk as others	127	33.2	-	-
Greater risk than other	60	15.7	-	-
COVID-19 knowledge score	-	-	6.8	2.5
Depressive symptoms (CES-D-10)	-	-	5.6	4.1

COVID-19, the coronavirus disease 2019; s.d., standard deviation; CES-D-10, 10-item Center for Epidemiologic Studies Depression.

### Demographic, health and socio-economic variables

During the Alert Level 5 lockdown (26 March 2020 to 30 April 2020) and the subsequent lifting to Alert Level 4 (beginning 01 May 2020), interviews were conducted through telephone in the preferred language(s) of the participant (e.g. isiZulu, isiXhosa, Sesotho or English) during the second wave of data collection. Research assistants conducted the interviews and translated the responses to English. All participants completed an extensive demographic survey, and household SES was assessed using an asset index that scored each participant according to the number of household physical assets that the participant possessed out of a possible 11 (e.g. electricity, fridge, stove or microwave, washing machine, satellite television, digital video disc [DVD] player, automobile, telephone, cell phone, computer and internet).

### Experiences of COVID-19 pandemic survey

A mixed-method survey was created during the weeks prior to the national lockdown and administered during the telephonic interviews. Our COVID-19 experience survey included a series of question that assessed the awareness of COVID-19, concern for COVID-19 infection, testing history and the impact of the pandemic on well-being. We also assessed the perceptions of COVID-19 prevention strategies, which asked whether a series of social and health behaviour practices was understood to prevent and decrease the risk of infection (e.g. can you get infected by being around people who cough and sneeze, sharing meals? Can you prevent transmission by wearing a face mask, social distancing, disinfecting surfaces, etc. to which the participants responded ‘yes’ or ‘no’.) We summed the number of correct answers to create a composite measure of ‘COVID-19 knowledge’ which included 12 items. The internal consistency of the knowledge score was 0.79. Perceived risk of COVID-19 infection was assessed by asking ‘Do you think you have the same risk as others?’ Participants indicated whether they had less, the same or more risk than the others.

### Psychological experiences

The 10-item Center for Epidemiologic Studies Depression (CES-D) scale assesses major symptoms of depression: depressed mood, changes in appetite and sleep, low energy, feelings of hopelessness, low self-esteem and loneliness. Respondents considered the presence and duration of each item or symptom over the past week and rated each along a 4-point scale from 0 (rarely or never) to 3 (most or all of the time). Possible scores range from 0 to 30: a score of 10 and above indicates the presence of significant depressive symptoms. We found the CES-D had an internal consistency of 0.78. At the end of each interview, we offered resources for free telephone-based psychological counselling at a major mental health non-governmental organisation in Johannesburg. Research assistants were encouraged to use these resources weekly because of the potential psychological burden of data collection.^19^

### Qualitative analyses

We collectively reviewed qualitative responses to open-ended questions and generated around 10 codes per question (*n* = 391). Then we systematically reviewed each response and attached codes to each response to reflect the themes in the response. Oftentimes, a single response was coded more than once. Then we (1) quantified these responses by using codes to reflect common variables and (2) explored the response to convey a deeper understanding of the perception of COVID-19 to understand how perceived risk and knowledge were framed. We compared responses by age, gender, education and household assets to describe the similarities and differences.

### Statistical analyses

All analyses were conducted using version 15.1 of Stata (Stata Corporation, College Station, TX). Chi-square tests of association with adjusted residuals, one-way analysis of variance (ANOVA) with Tukey *post hoc* tests and independent sample *t* tests were applied to categorical and continuous quantitative survey items, assessing the association with age (25‒44 years of age, 45+ years of age), sex (female, male), education (no school or primary school, secondary school or more) and household assets (sum of binary indicators of possession of electricity, fridge, stove or microwave, washing machine, satellite television, DVD player, automobile, telephone, cell phone, computer and internet).

All variables were examined for normal distribution and outliers. Bivariate analyses were conducted between COVID-19 knowledge score and predictors. Potential predictors of COVID-19 knowledge were identified through our qualitative research, as well as through past studies of other severe acute respiratory syndrome coronavirus 2 (SARS-CoV-2) virus infections and infectious respiratory diseases, including Middle East respiratory syndrome,^20^ severe acute respiratory syndrome,^21^ TB^22^ and COVID-19.^4,5^ With the exception of key demographic factors, only those that were statistically significant at the 0.1 level during bivariate analyses were included in the final models. The following variables were included in the final model: gender, age, SES, household density (inhabitants/rooms), coping ability, depressive symptom severity, concern about COVID-19 and perceived risk of COVID-19 infection. All covariates were assessed during the first wave of data collection aside from depressive symptom severity, concern about COVID-19 and perceived risk of COVID-19 infection. General psychosocial stress was evaluated for possible inclusion but was removed because of high covariance with the existing covariates. Self-reported quality of life and chronic illness status were considered but removed because their associations were not significant at the 0.1 level. Multiple ordinary least square (OLS) regression models were fitted to the data to estimate the correlates of COVID-19 knowledge.

### Ethical considerations

The University of the Witwatersrand Human Research Ethics Council reviewed and approved the study (M180544 and M190545); these ethics approvals were additional to the original ethics of the parent studies from which the cohort was invited, which facilitated rapport and a quick recruitment.

## Results

### Sample characteristics

The final sample size for this analytical sample was 391 adults. According to Table 1, the majority (71.8%) of the participants were female and between the ages of 25 and 44 (58.6%). Roughly half the sample (50.1%) had less than a secondary school education. All had heard of novel coronavirus and only 1.3% had been tested, and all had tested negative. Almost half of the sample (44.65%) perceived their risk to be lower than others.

### Qualitative perceptions, understandings and experiences of COVID-19

Table 2 and 3 presents the results from qualitative interview questions about participants’ knowledge and perceptions towards COVID-19 and experiences during the lockdown. Whilst most responses were short answers and oftentimes limited to a few words, we provide representative quotes to illustrate these perspectives in depth.

**TABLE 2:** Knowledge and perception of COVID-19.

Theme	Frequency	%
**What would you like to know about coronavirus?**		
I understand coronavirus and did not want to learn more about it	78	19.9
Helpful and effective treatments	41	10.5
How it spreads	31	7.9
Where and how to get tested or treated	28	7.2
When/if there will be a vaccine	27	6.9
How to protect themselves and their family	27	6.9
Where it came from or what causes it	18	4.6
What it is	18	4.6
When quarantine will end	13	3.3
What are the key symptoms	12	3.1
**What is the coronavirus?**		
A virus	210	53.7
A virus that kills	118	30.2
Flu or pneumonia	84	21.5
Airborne	69	17.6
It comes from or is related to the lung	58	14.8
Infectious	45	11.5
**Prevention methods utilised against COVID-19**		
Physical distancing	262	67.0
Hand washing	233	59.6
General hygiene	90	23.0
Face covering	49	12.5
Cleaning or disinfecting	23	5.9
Wearing gloves	21	5.4
Non-medical or herbal remedies	15	3.8
I don’t know	41	10.5

COVID-19, coronavirus disease 2019.

**TABLE 3:** Perception and experiences of COVID-19 and lockdown.

Theme	Frequency	%	Quote
**How did you feel when you first heard about COVID-19?**			
Scared or worried	92	23.5	I thought the virus would kill us.
Not serious	56	14.3	I never took it that serious.
Not affected	54	13.8	It would never come to South Africa.
**What do people in your community say about coronavirus?**			
Scared	184	47.1	Many are scared and complain about lockdown.
Denial	66	16.9	They say it does not exist.
Fatal	54	13.8	People in the community are scared, especially the older people, because we see the number of cases going up everyday. This virus kills.
Fear driving preventative behaviours	43	11.0	People are scared and practicing more preventative measures.
Unsure	39	10.0	I don’t know.
Non-compliance of regulations	32	8.2	People aren’t taking as seriously as they should.
It affects one’s life	20	5.1	People are starting to notice the seriousness of the virus.
**If you have other conditions, do you think coronavirus affects your other conditions?**			
No	176	45.0	No, I don’t think so.
Immune system	54	13.8	Yes, I think that coronavirus affects other conditions. I will quote an example with HIV. When a person with HIV gets the virus, their body is now dealing with two things thus the body’s defences will go down. The body will not be able to deal with fighting two different illnesses at once.
I don’t know	31	7.9	I’m unsure.
Yes	30	7.7	Yes, it does affect other conditions because by the time the person is infected, their immune system is already weak.
Chronic conditions	26	6.6	If someone with high blood pressure is infected with the virus, it could lower their blood pressure.
Lungs	24	6.1	Lungs, it enters through the nose and to the throat.
HIV	17	4.3	HIV people, because their immune system is weak.
Kidney disease	17	4.3	Yes kidney failure, if not detected earlier.
**Is COVID-19 similar to HIV?**			
No	327	83.6	HIV has a vaccine and COVID does not.
Worse	91	23.3	Not similar. It is worse than HIV and more deadly.
Similar	34	8.7	Yes, they are similar as the symptoms are similar as well.
**Do you have any other thoughts on coronavirus? How has coronavirus affected your life?**			
Financial stress	85	21.7	We can’t go out and work and make money so how will we have money to buy food, how will we live?
Social implications	26	6.6	We can’t even attend funerals of our friends and everything has stopped in our lives.
My life has not been affected	14	3.6	I am not affected, my life is still normal.
Food insecurity	8	2.0	As we are staying home during this time of the lockdown, I am concerned about how we will live. We can’t go out and work and make money so how will we get money to buy food, how will we live. They are giving out food parcels to only specific people. How is this fair when we are all not able to work, we are all in need of food. Why aren’t these food parcels for everyone, not just certain people?
General well-being	7	1.8	I’m always scared, every time I cough I think I am infected.
Religion	3	0.8	We need to repent and apologise to God, He will forgive us, our sins and heal us of this coronavirus.
Miscellaneous	3	0.8	I think if the Government can come to our townships and see how life is for themselves maybe they will understand the struggle.
			This time has forced us to think, re-evaluate, consider others. I think this is an ancestral thing, our ancestors are angry, black people have been oppressed for too long in this world, now our ancestors have brought this illness to bring the world on its knees, so that a new world order may begin, so that the black person may receive wealth. This period is more to bring us wealth than to take away our health.

COVID-19, coronavirus disease 2019; HIV, human immunodeficiency virus.

Most people described coronavirus as ‘a virus’ (210, 53.7%), or ‘it is a virus that kills’ (118, 30.2%).

Some were very specific, such as:

‘It is a virus that infects a person through the air and through germs. When you have it, the symptoms will show after 2 weeks. These include fever, tiredness, sweating and not sleeping enough.’ (Participant 193, 31-year old woman)

Others related it to flu or pneumonia (84, 21.5%), mentioned that it is airborne (69, 17.6%) and that it comes from or is related to the lung (58, 14.8%) or indicated that it is infectious (45, 11.5%). We also asked participants: ‘what would you like to know about coronavirus?’ Most participants indicated that they understood COVID-19 and did not want to learn more about it (78, 19.9%), but some wanted to know more about helpful treatments (41, 10.5%), and how it spreads (31, 7.9%), when will the vaccine be available or if there will be a vaccine at all for this disease (27, 6.9%). People defined ways you can get COVID as ‘touching your face’ (92, 23.5%), ‘I don’t know’ (88, 22.5%), exposure to someone with the virus, such as ‘being in close proximity to many people and sharing utensils’ (56, 14.3%), or ‘attending social gathering with many people’ or ‘being in a crowd’ (74, 18.9%). Others mentioned ‘not washing your hands’ (39, 10.0%).

When asked: ‘what do people in your community say about coronavirus?’, most indicated that they are scared (184, 47.1%), such as ‘many are scared and complain about lockdown’ and ‘they are scared’. Many indicated that many neighbours were in denial (66, 16.9%), such as ‘some don’t take it serious’ or ‘they say it does not exist’. Many said that neighbours feared it will kill them (54, 13.8%), such as ‘it will finish us all’ and ‘people in the community are scared, especially the older people, because we see the number of cases going up every day. This virus kills’. Washing hands (233, 59.6%) was the most common type of prevention methods reported, and it was frequently combined with other methods, such as ‘I wash my hands and stay home’ or ‘I wash my hands and wear a mask’ or ‘Constantly washing hands with soap, staying indoors most of the time, social distancing’. Most of these responses included some type of physical distancing comment (262, 67.0%), hygiene (90, 23.0%), wearing a face cover (49, 12.5%), cleaning or disinfecting (23, 5.9%) and wearing gloves (21, 5.4%). Some mentioned non-medical or herbal remedies (15, 3.8%) such as prayer or ‘I drink hot liquid, one lemon a day, wash hands and avoid crowded areas’.

Additionally, in response to the question: ‘If you have other conditions, do you think coronavirus affects your other conditions?’. Most said ‘no’ (176, 45.0%) or ‘I don’t know’ (31, 7.9%). For those who said ‘yes’ (30, 7.7%), we asked ‘How?’ For example, one person said, ‘Yes, I think that coronavirus affects other conditions’:

‘I will quote an example with HIV. When a person with HIV gets the virus, their body is now dealing with two things; thus, the body’s defences will go down. The body will not be able to deal with fighting two different illnesses at once.’ (Participant 23, 35-year old woman)

Some participants said susceptibility was associated with ‘HIV people because their immune system is weak’, with 54 participants (13.8%) mentioning a weak immune system and many linking it to HIV (17, 4.3%) or chronic conditions (26, 6.6%).

Finally, we asked participants: ‘do you have any other thoughts on coronavirus? How has coronavirus affected your life?’ Participants discussed various impacts on their life, including increased financial stress (85, 21.7%) or food insecurity (8, 2.0%). For instance, one participant states: ‘we can’t go out and work and make money so how will we get money to buy food, how will we live’. Many also spoke about the social implications of the virus (26, 6.6%), for example: ‘I’m unable to socialise to relieve my stress’ or ‘we can’t even attend funerals of our friends and everything has stopped in our lives’. Notably, some felt their lives had not been affected (14, 3.6%), whilst others declined the question (133, 34.0%). Others spoke about religious implications of the virus (3, 0.8%): ‘we need to repent and apologise to God, He will forgive us, our sins and heal us of this coronavirus’. A few spoke about the controversies surrounding the virus (3, 0.7%), for example: ‘yes, there are rumours that this virus is manmade, particularly by the Chinese for the sake of population control. Yet governments around the world deny such theories’.

Finally, a few participants spoke about their implications of the virus for socio-economic or racial justice.

For example, ‘I think if the Government can come to our townships and see how life is for themselves maybe they will understand the struggle’ and:

‘This time has forced us to think, re-evaluate, consider others. I think this is an ancestral thing, our ancestors are angry, black people have been oppressed for too long in this world, now our ancestors have brought this illness to bring the world on its knees, so that a new world order may begin, so that the black person may receive wealth. This period is more to bring us wealth than to take away our health.’ (Participant 209, 29-year old woman)

Complete results from the qualitative interviews are listed in Table 2 and 3.

### Quantitative data – Descriptive statistics

Table 4 summarises that the common forms of perceived transmission reported were being around people who sneeze (79.5%) or cough (55.0%) and touching others (50.6%). The most commonly perceived comorbid risk of COVID-19 was reported as TB (68.8%), followed by HIV (57.0%), and diabetes (39.4%). Most frequently mentioned methods for prevention were hand washing with soap and water (89.5%), staying home (67.0%) and covering your mouth when coughing or sneezing (47.8%). The majority of the samples was concerned (38.1%) or very concerned (46.5%) about COVID-19.

**TABLE 4:** Subgroup differences amongst quantitative survey items (*n* = 391).

Survey item	Overall sample		Age				Gender				Education				Household Assets	
			25–44 (*n* = 225)		45+ (*n* = 159)		Female(*n* = 78)		Male (*n* = 109)		No school or primary school (*n* = 194)		Secondary school or more (*n* = 193)		Continuous (*n* = 391)	
	*N*	%	*N*	%	*N*	%	*N*	%	*N*	%	*N*	%	*N*	%	Mean	s.d.
**How do you get COVID?**																
Being around people who cough	215	55.0	**136**	60.40*	76	47.80*	155	55.8	59	54.10	89	45.90**	**125**	64.80**	7.98	1.84
Being around people who sneeze	311	79.5	185	82.20	122	76.70	223	80.2	86	78.90	150	77.30	159	82.40	7.84	1.80
Receiving a curse	9	2.3	7	3.10***	1	0.60***	7	2.5	2	1.80	1	0.50*	**8**	4.20*	7.89	1.45
Sharing meals	45	11.5	**37**	16.40**	7	4.40**	35	12.6	10	9.20	5	2.58**	**40**	20.73**	8.13	1.98
Touching others	198	50.6	122	54.20	73	45.90	144	51.8	52	47.70	79	40.70**	**117**	60.60**	8.03	1.74***
Don’t know/unsure	7	1.8	3	1.30	4	2.50	5	1.8	2	1.80	4	2.10	3	1.60	7.00	1.73
**Are you at greater risk of COVID if you have?**																
Cancer	81	20.7	**62**	27.60**	18	11.30**	59	21.2	22	20.20	19	9.80**	**62**	32.10**	**8.36**	1.80**
Diabetes	154	39.4	95	42.20	57	35.90	**119**	42.8*	34	31.20*	65	33.50*	**88**	45.60*	7.95	1.87
Drinking alcohol	69	17.7	53	23.60**	14	8.80**	56	20.1***	13	11.90***	16	8.30**	**53**	27.50**	**8.28**	1.85*
Experiencing poverty	59	15.1	43	19.10**	15	9.40**	41	14.8	18	16.50	16	8.30**	**43**	22.30**	8.25	1.78***
High blood pressure	75	19.2	54	24.00**	20	12.60**	**61**	21.9*	14	12.80*	17	8.80**	**58**	30.10**	8.20	1.97***
HIV	223	57.0	137	60.90	84	52.80	162	58.3	60	55.10	101	52.10*	**121**	62.70*	**8.04**	1.75*
Malnutrition	57	14.6	**45**	20.00**	11	6.90**	44	15.8	13	11.90	10	5.20**	**47**	24.40**	**8.42**	1.95*
Mental illness	39	10.0	**34**	15.10**	4	2.50**	33	11.9***	6	5.50***	4	2.10**	**35**	18.10**	**8.72**	1.72**
Stress	45	11.5	32	14.20***	13	8.20***	36	13.0	9	8.30	14	7.20**	**31**	16.10**	8.36	2.01***
Tuberculosis	269	68.8	154	68.40	110	69.20	192	69.1	74	67.90	128	66.00	138	71.50	7.96	1.84
Other diseases or social conditions	25	6.4	**20**	8.90*	5	3.10*	20	7.2	5	4.60	5	2.60**	**20**	10.40**	**8.87**	1.28*
Don’t know/unsure	66	16.9	39	17.30	26	16.40	44	15.8	21	19.20	35	18.00	30	15.50	7.74	1.67
**What will help prevent coronavirus transmission?**																
Ancestors	3	0.8	3	1.30	0	-	3	1.1	0	-	0***	-	3	1.60***	9.33	1.15
Covering your mouth when coughing/sneezing	187	47.8	108	48.00	76	47.80	137	49.3	49	45.00	89	45.90	97	50.30	7.89	1.79
Disinfecting surfaces	143	36.6	84	37.30	55	34.60	100	36.0	42	38.50	59	30.40*	**83**	43.00*	8.01	1.83
Drinking water	72	18.4	**50**	22.20*	21	13.20*	53	19.1	19	17.40	27	13.90*	**45**	23.30*	8.00	1.91
Eating healthy foods	60	15.4	**44**	19.60**	15	9.40**	43	15.5	17	15.60	19	9.80**	**41**	21.20**	8.15	1.69
Exercise	38	9.7	**32**	14.20**	**5**	3.10**	28	10.1	10	9.20	7	3.60**	**31**	16.10**	8.13	2.02
Going to a sangoma or traditional healer	2	0.5	1	0.40	1	0.60	1	0.4	1	0.90	1	0.50	1	0.50	7.00	1.41
Going to church	6	1.5	2	0.90	3	1.90	3	1.1	3	2.80	3	1.60	3	1.60	8.00	1.26
Hand washing only with water	20	5.1	**17**	7.60*	3	1.90*	15	5.4	5	4.60	4	2.10**	**16**	8.30**	7.55	1.61
Hand washing with water and soap	350	89.5	197	87.60	146	91.80	247	88.9	99	90.80	17	90.20	171	88.60	7.87	1.82
Having faith in God	22	5.6	**18**	8.00**	3	1.90**	18	6.5	4	3.70	4	2.10**	**18**	9.30**	8.13	1.61
Having someone pray for you	4	1.0	3	1.30	0	-	3	1.1	1	0.90	0*	-	**4**	2.10*	8.75	1.26
Prayer	59	15.1	37	16.40	21	13.20	45	16.2	14	12.80	27	13.90	32	16.60	8.03	1.78
Physical distancing	143	36.6	87	38.67	53	33.30	101	36.3	40	36.70	58	29.90**	**83**	43.00**	**8.13**	1.85*
Staying home	262	67.0	**161**	71.60*	95	59.80*	190	68.4	68	62.40	115	59.30**	**143**	**74.10**	**8.02**	1.73*
Taking vitamins	60	15.4	**45**	20.00**	14	8.80**	46	16.6	14	12.80	16	8.30**	**44**	22.80**	**8.38**	1.71*
Wearing a face cover	110	28.1	68	30.20	41	25.80	75	27.0	34	31.20	44	22.70*	**65**	33.70*	**8.15**	1.89*
Other	6	1.5	6	2.70*	0	-	3	1.1	3	2.75	1	0.50***	5	2.60***	7.33	3.01
Don’t know/unsure	4	1.0	3	1.30	1	0.60	4	1.4	0	-	2	1.00	2	1.00	7.00	1.41
**How concerned are you about coronavirus?**																
Very unconcerned	9	2.4	**9**	4.20*	0	0.00*	8	3.0	1	0.90	1	0.50**	**8**	4.30**	7.67	1.58
Unconcerned	13	3.4	9	4.20	4	2.50	9	3.4	4	3.70	3	1.60**	**10**	5.40**	7.62	2.18
Neutral	37	9.7	20	9.20	16	10.10	16	6.0**	**21**	19.40**	18	9.50	19	10.20	8.22	1.90
Concerned	145	38.1	73	33.60*	**70**	44.30*	105	39.0	38	35.20	**89**	46.80**	54	28.90**	7.47	1.79**
Very concerned	177	46.5	106	48.90	68	43.00	131	48.7	44	40.70	79	41.60	96	51.30	**8.14**	1.73**

COVID, coronavirus disease; s.d., standard deviation; HIV, human immunodeficiency virus.

* , denotes significance at *p* < 0.05 level;

** , denotes significance at *p* < 0.01 level;

*** , denotes significance at *p* < 0.10 level.

†, Subgroup differences amongst demographic categorical variables (age, sex and education) assessed using Pearson’s chi-square test of association. Differences amongst continuous variables (household assets) assessed using one-way ANOVA and Tukey *post hoc* tests as well as independent sample *t* tests.

‡, Percentages may not add to 100 because of the ability of participants to make responses in more than one category.

§, Adjusted residuals greater or lesser than absolute value of 2 were used to determine where significant differences occurred following significant chi-square tests. Tukey *post hoc* testing was performed to determine where significant differences occurred following significant ANOVA tests.

¶, *p* values are uncorrected (no penalty induced for the number of tests performed).

Boldface indicates groups with higher than expected frequencies of response in the indicated category.

Table 5 indicates that age, education and wealth played a role in perceived risk and concern for COVID-19, whilst gender did not. Those who were older (45+) and had completed fewer years of school were more likely to report being ‘very concerned’ or ‘concerned’, and those who had more assets were more likely to report being ‘very concerned’ about COVID-19. Although older adults (45+) were more likely to be concerned about COVID-19, older adults were less likely to report perceived risk factors for COVID-19 such as exposure to cough or sharing meals and also were less likely to report cancer, drinking alcohol, poverty and high blood pressure as increasing the COVID-19 risk. Older individuals are also less likely to mention drinking water, eating healthy foods and exercise, as the key forms for prevention of COVID-19. Moreover, those with a secondary school education or more reported frequently perceived risk associated with exposure to cough, shared meals and touching others, whereas less educated people (no school or primary school) reported more concern about contracting the disease. Individuals with more education and more assets prioritised prevention methods such as disinfecting surfaces, physical distancing, staying home and wearing a face cover. Table 4 displays the percentage of people who correctly identified the effective prevention practices against COVID-19.

**TABLE 5:** Predictors of coronavirus disease 2019 knowledge.

Variable	*β*	s.d.
Gender (Female)	0.2300	0.30
Age	−0.1600	0.01
Education	0.2400	0.10***
Asset	0.0920	0.08
Density	−0.1500	0.10
Coping	0.0100	0.01
COVID-19 risk	−0.5000	0.20**
Depression	0.0800	0.03*
Concern	0.600-	0.20**
Intercept	2.4000	1.50**
Model *R*^2^	0.1380	-

COVID-19, coronavirus disease 2019; s.d., standard deviation.

* , *p* < 0.05;

** , *p* < 0.01;

*** , *p* < 0.10.

### Quantitative data – Regression analyses

Complete data for the regression analyses were available for 234 participants. Table 5 presents the results of the OLS regression analyses of demographic, household and psychological factors that were correlated with COVID-19 knowledge. Greater concern about COVID-19 (*p* = 0.001) and more severe depressive symptoms (*p* = 0.015) were associated with greater COVID-19 knowledge. Additionally, greater perceived risk of COVID-19 infection was negatively associated with COVID-19 knowledge (*p* = 0.001). Higher educational attainment was positively associated with greater COVID-19 knowledge, although the estimated association was not statistically significant (*p* = 0.076). Age, household density, SES and coping ability were not statistically significant correlates of COVID-19 knowledge.

## Discussion

This study of 391 Soweto residents amidst the first 6 weeks of the South African national lockdown, or Level 5 lockdown, demonstrates that the perceptions and knowledge of COVID-19 varied across several demographic characteristics. Our findings also showed moderate levels of understanding about COVID-19, prevention methods and risk as well as exposure to major physical, psychosocial and financial stressors. Finally, in our quantitative analyses, we found that worse depressive symptoms, lower perceived infection risk and greater concern about COVID-19 were correlated with higher COVID-19 prevention knowledge. These findings are similar to the estimates published by Reddy and colleagues,^6^ who surveyed 55 823 participants at the end of March 2020 and found that 94.2% of their sample indicated that coughing and sneezing were the mode of transmission for the virus and 25.0% perceived themselves at high risk of the virus, compared with 55.0% in our study who believed coughing was a mode of transmission, 79.5% who believed sneezing was a mode of transmission and 15.7% who perceived themselves to be at greater risk than others for infection. The relatively smaller proportion of study participants in our study who endorsed these beliefs could be the result of the differences in the two study samples: Reddy and colleagues conducted an online national survey of a relatively heterogenous group of individuals across South Africa, whilst our study was conducted through telephone and specifically enrolled residents of Soweto, representing a sample with diverse demographic and cultural characteristics yet limited to a smaller geographic area.

The knowledge level of participants in our study with respect to COVID-19 appears to substantially exceed that of other emerging health issues in the country such as breast cancer.^23^ The knowledge base for COVID-19 was comparable to that of HIV amongst Sowetan adults, as indicated by Nachega and colleagues’ study of HIV-infected adults in Soweto (*n* = 105).^24^ They found that 83% of adults reported knowing about modes of transmission for HIV.^24^ This high level of awareness of COVID-19 may reflect the success of widespread public health education campaigns as well as an output of the far-reaching impact of the virus on social and economic activities. Despite these overall positive findings, the results still present some cause for concern with respect to COVID-19 knowledge. For instance, at the time the study was conducted, only 11.5% reported that sharing a meal is a method for transmission, less than half (47.8%) agreed that transmission could be prevented by covering one’s mouth whilst coughing and sneezing and only 28.1% agreed that transmission could be prevented through wearing a face cover. Moreover, the descriptive analyses suggest that knowledge of COVID-19 on specific items differs as a function of age, education and household assets, although it is important to note that age and SES were not significantly associated with the composite COVID-19 knowledge score in multivariable analyses. Furthermore, although participants reported actively engaging in various prevention methods for the virus, they were also aware of some factors which they cannot change, such as living in close quarters, being unable to afford personal protective equipment and having pre-existing conditions such as HIV, diabetes or psychosocial challenges; awareness of these issues may introduce aspects of fatalism or increased anxiety in this population’s response to the pandemic.

Importantly, in our study, we were able to estimate the associations between the severity of depressive symptoms, perceived risk and personal concern about COVID-19 and knowledge of COVID-19. Our findings suggest an interesting link between prior indications of depressive symptoms and increased knowledge of COVID-19, perhaps pointing to the propensity of individuals who are depressed to concentrate on negative aspects of their lives, including knowledge about specific disease risks.^2,25,26^ Similarly, and perhaps in support of this line of thinking, we also observed an association between higher concurrent levels of concern and greater levels of knowledge about the virus. Finally, we observed that an individual’s perception of risk was associated with lower knowledge scores about the virus. This elevated level of risk could be the result of fearmongering about the virus that has taken place in the general media, perhaps leading those receiving some information but not extensive information about the virus to overestimate its threat. Overall, these findings are important to tease out with further research, given their implications for not only COVID-19 awareness and knowledge but also the understanding of the pathways between perceptions of risk and mental health and knowledge of a health risk, which may be an important factor in the progression of other major health threats.

One strength of this study in comparison with other studies on knowledge and awareness of COVID-19 in South Africa^6,13^ is that participants were interviewed directly by research assistants in their local languages, generating richer qualitative data than is possible with other data collection methods such as larger-scale surveys. In doing so, we were able to ascertain some of the non-health-related impacts of the COVID-19 pandemic on our study population. These include extreme financial stress, impacts on social support, increased levels of worry and anxiety and feelings of their lives being ‘on pause’. As the second wave of COVID-19 has erupted in most of the world, policymakers are attempting to balance the risk of increased or uncontrollable infection rates as a result of COVID-19 and the consequences of COVID-19-related restrictions on people’s well-being and quality of life. Although South Africa is not positioned with the resources to cope with a large number of severe COVID-19 cases, the country is also faced with the challenge of a population that will considerably suffer from the economic effects of shutdowns.

The limitations of our study include that the sample size is relatively small, and that it is unknown how generalisable the findings are to other geographical contexts in South Africa. Furthermore, the participants were recruited by telephone, which required individuals to have a cell phone and complete the interview during the normal working hours. These components of data collection may perhaps have excluded those from lower socio-economic backgrounds who may not possess cell phones or households living in regions with poor network coverage and electricity outages, which were common in Soweto during the lockdown (Buys et al. 2009; Dalvit et al. 2014; Kim et al. 2020). Despite this, we believe the study offers both some interesting findings related to the relationships between the severity of depressive symptoms, concern, perceived risk and COVID-19 knowledge and nuanced insight into the perceptions Sowetan residents have of the pandemic and its impact on their lives, which may assist policymakers moving forward.

## Conclusion

This descriptive study demonstrates moderate levels of understanding of COVID-19 transmission and COVID-19 risk amongst a concentrated sample of Sowetan residents in an area with a high burden of disease and difficult barriers to care. Public health campaigns should focus on continuing to improve knowledge about preventative behaviours and reduce misinformation associated with the virus as well as target-specific subgroups of the population who have been identified as having lower levels of knowledge about the disease (see Reddy et al. 2020). Policymakers around the world and in South Africa should consider the mental health and non-health-related impact of the pandemic on their citizens in order to curb the pandemic in a way that maximises the well-being as much as possible.

## References

[1] Asiamah N, Opuni FF, Mends-Brew E, Mensah SW, Mensah HK, Quansah F. Short-term changes in behaviors resulting from COVID-19-related social isolation and their influences on mental health in Ghana. Community Ment Health J. 2021;57(1):79–92. 10.1007/s10597-020-00722-4 33033971 PMC7543965

[2] Kim AW, Nyengerai T, Mendenhall E. Evaluating the mental health impacts of the COVID-19 pandemic: Perceived risk of COVID-19 infection and childhood trauma predict adult depressive symptoms in urban South Africa. Psychol Med. 2020;8. 1–13. 10.1017/S0033291720003414 PMC7520640 32895082

[3] Sediri S, Zgueb Y, Ouanes S, et al. Women’s mental health: Acute impact of COVID-19 pandemic on domestic violence. Arch Womens Ment Health. 2020;23(6):749–756. 10.1007/s00737-020-01082-4 33068161 PMC7568008

[4] Baig M, Jameel T, Alzahrani SH, et al. Predictors of misconceptions, knowledge, attitudes, and practices of COVID-19 pandemic among a sample of Saudi population. PLoS One. 2020;15(12):e0243526. 10.1371/journal.pone.0243526 33296420 PMC7725365

[5] Naser AY, Dahmash EZ, Alwafi H, et al. Knowledge and practices towards COVID-19 during its outbreak: A multinational cross-sectional study. MedRxiv. 2020. 10.1101/2020.04.13.20063560

[6] Reddy SP, Sewpaul R, Mabaso M, et al. South Africans’ understanding of and response to the COVID-19 outbreak: An online survey. S Afr Med J. 2020;110(9):894–902. 10.7196/SAMJ.2020.v110i9.14838 32880275

[7] Strecher VJ, Rosenstock IM. The health belief model. In: Connor M, Norman P, editors. Cambridge handbook of psychology, health and medicine. Two Penn Plaza, NY: McGraw-Hill, 1997; p. 113, 117.

[8] Shamu S, Khupakonke S, Farirai T, et al. Knowledge, attitudes and practices of young adults towards HIV prevention: An analysis of baseline data from a community-based HIV prevention intervention study in two high HIV burden districts, South Africa. BMC Public Health. 2020;20(1):1249. 10.1186/s12889-020-09356-3 32807116 PMC7433171

[9] Shushtari ZJ, Hosseini SA, Sajjadi H, Salimi Y, Shahesmaeili A, Snijders TA. HIV risk perception and sexual behaviors among female sex workers in Tehran, Iran. Med J Islam Repub Iran. 2019;33(1):101. 31934561 10.34171/mjiri.33.101 PMC6946931

[10] Anand A, Shiraishi RW, Bunnell RE, et al. Knowledge of HIV status, sexual risk behaviors and contraceptive need among people living with HIV in Kenya and Malawi. AIDS. 2009;23(12):1565–1573. 10.1097/QAD.0b013e32832cb10c 19542867

[11] Kincaid DL, Babalola S, Figueroa ME. HIV communication programs, condom use at sexual debut, and HIV infections averted in South Africa, 2005. J Acquir Immune Defic Syndr. 2014;66(Suppl 3):S278–S284. 10.1097/QAI.0000000000000242 25007197

[12] Peltzer K, Parker W, Mabaso M, Makonko E, Zuma K, Ramlagan S. Impact of national HIV and AIDS communication campaigns in South Africa to reduce HIV risk behaviour. Sci World J. 2012;2012:384608. 10.1100/2012/384608 PMC3504395 23213285

[13] Burger R, Christian C, Maughan-Brown B, Rensburg R, Rossouw L. COVID-19 risk perception, knowledge and behaviour. National Income Dynamics Study (NIDS) – Coronavirus Rapid Mobile Survey (CRAM); 2020.

[14] Karim SSA, Churchyard GJ, Karim QA, Lawn SD. HIV infection and tuberculosis in South Africa: An urgent need to escalate the public health response. Lancet. 2009;374(9693):921–933. 10.1016/S0140-6736(09)60916-8 19709731 PMC2803032

[15] Oni T, Youngblood E, Boulle A, McGrath N, Wilkinson RJ, Levitt NS. Patterns of HIV, TB, and non-communicable disease multi-morbidity in peri-urban South Africa – A cross sectional study. BMC Infect Dis. 2015;15(1):1–8. 10.1186/s12879-015-0750-1 25595711 PMC4300166

[16] Alexander P, Ceruti C, Motseke K, Phadi M, Wale K. Class in Soweto. Scottsville: University of KwaZulu-Natal Press; 2013.

[17] Richter LM, Mathews S, Kagura J, Nonterah E. A longitudinal perspective on violence in the lives of South African children from the birth to twenty plus cohort study in Johannesburg-Soweto. S Afr Med J. 2018;108(3):181–186. 10.7196/SAMJ.2017.v108i3.12661 30004360

[18] Coovadia H, Jewkes R, Barron P, Sanders D, McIntyre D. The health and health system of South Africa: Historical roots of current public health challenges. Lancet. 2009;374(9692):817–834. 10.1016/S0140-6736(09)60951-X 19709728

[19] Kim AW. Promoting mental health in community and research settings during COVID-19: Perspectives and experiences from Soweto, South Africa. Am J Hum Biol. 2020;32(5):e23509. 10.1002/ajhb.23509 32978877 PMC7831381

[20] Balaban V, Stauffer W, Hammad A, et al. Predictors of protective behaviors among American travelers to the 2009 Hajj. J Epidemiol Glob Health. 2013;3(4):187–196. 10.1016/j.jegh.2013.08.001 24206790 PMC7320419

[21] Cheng C, Ng AK. Psychosocial factors predicting SARS-preventive behaviors in four major SARS-affected regions. J Appl Soc Psychol. 2006;36(1):222–247. 10.1111/j.0021-9029.2006.00059.x

[22] Naidoo P, Simbayi L, Labadarios D, et al. Predictors of knowledge about tuberculosis: Results from SANHANES I, a national, cross-sectional household survey in South Africa. BMC Public Health. 2016;16(1):1–12. 10.1186/s12889-016-2951-y 26987759 PMC4797251

[23] Maree JE, Wright SC. How would early detection be possible? An enquiry into cancer related knowledge, understanding and health seeking behaviour of urban black women in Tshwane, South Africa. Eur J Oncol Nurs. 2010;14(3):190–196. 10.1016/j.ejon.2009.10.009 19944646

[24] Nachega JB, Lehman DA, Hlatshwayo D, Mothopeng R, Chaisson RE, Karstaedt AS. HIV/AIDS and antiretroviral treatment knowledge, attitudes, beliefs, and practices in HIV-infected adults in Soweto, South Africa. J Acquir Immune Defic Syndr. 2005;38(2):196–201. 10.1097/00126334-200502010-00011 15671805

[25] Lueck JA. What’s the risk in seeking help for depression? Assessing the nature and pleasantness of outcome perceptions among individuals with depressive symptomatology. Health Educ Behav. 2019;46(3):463–470. 10.1177/1090198118811898 30409048

[26] Sokol Y, Serper M. Temporal self appraisal and continuous identity: Associations with depression and hopelessness. J Affect Disord. 2017;208:503–511. 10.1016/j.jad.2016.10.033 27832930

